# Conservation Genomics of a Threatened *Rhododendron*: Contrasting Patterns of Population Structure Revealed From Neutral and Selected SNPs

**DOI:** 10.3389/fgene.2020.00757

**Published:** 2020-09-04

**Authors:** Detuan Liu, Lu Zhang, Jihua Wang, Yongpeng Ma

**Affiliations:** ^1^Yunnan Key Laboratory for Integrative Conservation of Plant Species with Extremely Small Populations, Kunming Institute of Botany, Chinese Academy of Sciences, Kunming, China; ^2^Flower Research Institute, Yunnan Academy of Agriculture Sciences, Kunming, China

**Keywords:** *Rhododendron cyanocarpum*, ddRAD-seq, genetic diversity, population demography, conservation

## Abstract

Though it is well-acknowledged that next generation sequencing (NGS) technologies can provide further insights into plant conservation management than traditional molecular markers, studies employing NGS to address conservation genomics and subsequent conservation strategies for threatened plants are still rare. *Rhododendron* is the largest genus of woody plants in China, and many species are threatened, however, to date there has been no conservation genetic research using NGS in this genus. In the present study, we investigated the conservation genetics of *R. cyanocarpum*, a threatened species endemic to the Cangshan Mountains in Yunnan, China, using a double digest restriction-site-associated DNA-sequencing (ddRAD-seq) approach. Due to the availability of sufficient SNPs, we were able to distinguish between neutral and putatively selected SNPs and were able to further investigate the genetic diversity, population structure, and differentiation in *R. cyanocarpum*, as well as make an estimation of its demographic history. A total of 6,584 SNPs were obtained, of which 5,729 were neutral (detected using Tajima’s D). In terms of the 5,729 neutral SNPs, *R. cyanocarpum* had a higher genetic diversity (π = 0.0702 ± 0.0017, *H_*e*_* = 0.0675 ± 0.0016) than other plant species assessed using Rad-seq methods, while population differentiation (*F*_*st*_ from 0.0314 to 0.0452) was weak. Interestingly, contrasting patterns of population structure were revealed from all neutral and selected SNPs, with distinct genetic clusters forming for all SNPs and neutral SNPs, but no distinct subgroups for selected ones. Moreover, we were able to detect changes in effective population size (*N*_*e*_) of *R. cyanocarpum* from 150,000 years ago, including a bottleneck event ca. 60,000 years ago, followed by recovery of *N*_*e*_ over a short period, and a subsequent gradual decline in *N*_*e*_ to date. Implications for conserving *R. cyanocarpum* based on these main results are then discussed.

## Introduction

Biodiversity conservation is key in the field of conservation, and the essence of all biodiversity is genetic variation (Culver et al., 2011). Indeed, one can argue that the conservation of biodiversity is ultimately the conservation of genetic diversity. Genetic diversity strongly influences the ability of plant species to persist in the face of threats (Hendricks et al., 2017), and loss of genetic diversity has been considered as a crucial factor that results in inbreeding depression, reduced adaptation and fitness, and a decrease in long-term species survival (Bruni et al., 2012). Endangered species with small isolated populations are at elevated risk of losing adaptive variation due to genetic drift and the genetic costs of inbreeding (Allendorf et al., 2007). It is therefore important to pay attention to the genetic diversity, genetic structure, demographic history, and genetic background of an endangered species before developing protection measures (Wang and Hu, 1996; Wu et al., 2017; Barbosa et al., 2018; Li et al., 2018). Understanding the extent of intraspecific genetic diversity and population genetic structure is important for planning conservation strategies for endangered species, and it is often necessary to investigate the causes of low levels of genetic variation in populations, especially for plant species with extremely small populations.

Population genetics, when based on a relatively wide distribution, can provide a rich and mathematically rigorous framework for understanding evolutionary processes in natural populations. Common marker types, such as microsatellites or AFLP, can only generate limited markers per sample (Hodel et al., 2016). With high-throughput sequencing technological advances, generally called Next Generation Sequencing (NGS), population genomics can now address evolutionary processes at a genomic scale in natural populations with thousands of genetic markers rather than a few genetic loci (Hohenlohe et al., 2010). Restriction site-associated DNA sequencing (RAD-seq), based on Illumina NGS technology, is relatively cheap and flexible, and has fueled studies in conservation genomics (Andrews et al., 2016). This has led to the discovery and genotyping of thousands of polymorphic genetic markers in non-model species, and the technique has now become a popular tool for the study of the selection and genetics of adaptation in natural populations (Andrews et al., 2016; Catchen et al., 2017). In addition, in datasets comprising fewer individuals, RAD-seq tends to outperform microsatellites, giving more reliable inferences on population structure and a higher resolution (Lemopoulos et al., 2019).

The genus *Rhododendron* L. (Ericaceae) contains more than 1,000 species and has a global distribution (Chamberlain et al., 1996). To date, a total of ca. 600 species are found in China (Ma, 2015; Tian et al., 2019), of which 70% are endemic and many are threatened, so the conservation of *Rhododendron* is urgent (Ma et al., 2014; Qin et al., 2017). Although there have been some reports on the population genetics of *Rhododendron*, few of these studies focused on threatened species (Ma et al., 2017). Furthermore, we are unaware of any population genetic studies employing RAD-seq methods in *Rhododendron*.

*Rhododendron cyanocarpum* Franch. ex W. W. Smith, Subgen. *Hymenanthes* (Blume) K. Koch and Subsect. *Thomsonii* Sleumer, is a threatened species endemic to the Cangshan Mountains in Dali, Yunnan Province, Southwest China (Zhang et al., 1998). *R. cyanocarpum* has four populations growing along the ridges of the Cangshan Mountains, and is classified as Vulnerable in the IUCN red list (World Conservation and Monitoring Centre, 1998), in the Red list of Rhododenrons (Gibbs et al., 2011) and in the Threatened Species List of China’s Higher Plants (Qin et al., 2017). *R. cyanocarpum* grows at altitudes of 3,400–3,900 m; 12 individuals were found at a 900 m^2^ plot (Zhang et al., 1998). Its main effective pollinators are bumblebees (Ma et al., 2015a), with a 10-km potential range (Goulson and Stout, 2001) and at least 1.5 km foraging range from their colonies (Osborne et al., 2008). In addition, birds might be another pollinator in the late flowering of *R. cyanocarpum* (Ma et al., 2015a). Temperature is a crucial factor influencing the survival rate of its seedlings (Zhang et al., 1998). Furthermore, polymorphism of flower color (Ma et al., 2015b), reproductive isolation, and natural hybridization were also reported (Ma et al., 2010a, b, 2016; Ma, 2010). However, to date, no study has investigated the population genetics of *R. cyanocarpum*. Here we hypothesized that, due to very similar habitats where *R. cyanocarpum* occurs, adaptive selection could tend to be harmonized among populations. Therefore, the population structure of *R. cyanocarpum* inferred from selected loci (if some can be detected) should show more similar patterns than neutral ones. Moreover, we supposed that historical demography (e.g., bottleneck) could play an important role in maintaining the current distribution of *R. cyanocarpum*.

In our study, we assessed the genetic diversity of *R. cyanocarpum* using ddRAD-seq (Peterson et al., 2012). Due to the availability of sufficient SNPs, we were able to investigate several aspects of the population genetics of *R. cyanocarpum* using neutral and putatively selected SNPs, including genetic diversity, population structure, and differentiation, as well as estimation of demographic history. We believe that both the analytical process and the results presented here will be useful in the future, not only for the management and conservation of *R. cyanocarpum*, but also as an example of population genetic studies employing NGS for the conservation of endangered plants, including the new conservation action concept of plant species with extremely small populations (PSESP) in China (Ma et al., 2013; Sun, 2013; Sun et al., 2019).

## Materials and Methods

### Sample Collection and Extraction of DNA

**FIGURE 1:**
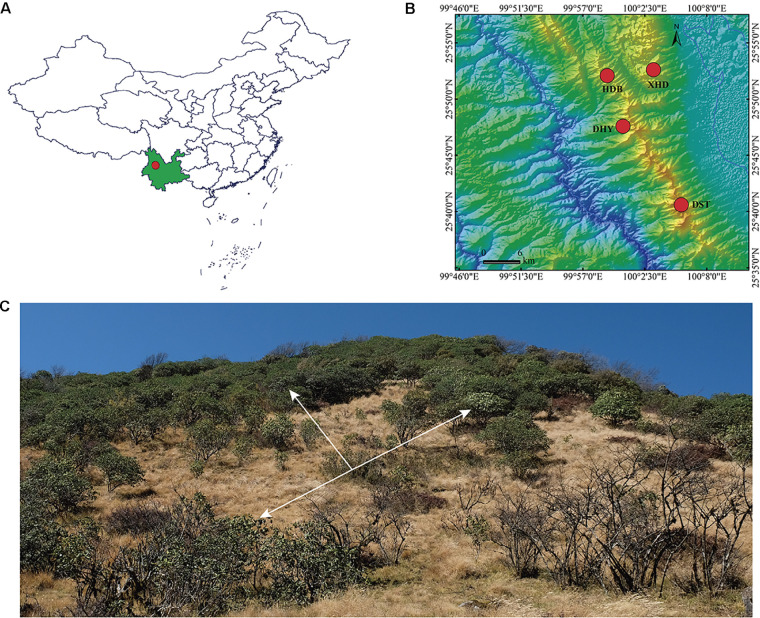
Distribution and habitat of *R. cyanocarpum*. **(A)** Dali (red dot) in Yunnan Province (green), southwestern China; **(B)** population distribution of *R. cyanocarpum* on the Cangshan Mountains; **(C)** habitat, the white arrows indicate plants of *R. cyanocarpum*.

*Rhododendron cyanocarpum* is endemic to the Cangshan Mountains, Yunnan, southwestern China. We sampled 59 individuals from four populations at locations around the Cangshan Mountains (Figure 1 and Table 1). Within each population, individuals spaced about 50 m apart were randomly selected. Leaf material was collected into silica gel in the field. Total genomic DNA was then extracted from the silica gel-dried leaf material using a CTAB procedure (Doyle and Doyle, 1990). In brief, gel-dried fragments of samples were put into liquid nitrogen and quickly ground to powder; the powder was then immediately transferred into a clean 1.5 ml microcentrifuge tube and mixed immediately with 700 μl 2 × CTAB extracting solution and incubated in a water bath at 65°C for 30 min. The supernatant was then removed, and the pellet was resuspended in chloroform-isoamyl alcohol (24:1). The samples were then centrifuged at 12,000 rpm, after which the supernatant was transferred to another clean 1.5 ml microcentrifuge tube and mixed with the same volume of isopropanol, then incubated at −20°C for about 1 h. The solution was then centrifuged at 12,000 rpm for 10 min, then the supernatant liquor was carefully discarded. The DNA was then cleaned twice with 75% ethanol and air dried at room temperature. The cleaned DNA was then dissolved in 50 μl TE, and 0.5 μl RNase was added to digest RNA. DNA quantity was assessed with 1.2% agarose gel electrophoresis and quantified with a qubit 3.0 fluorescent quantitative assay. DNA extraction and ddRAD-seq library preparation were performed by JieRui BioScience Co. Ltd. (Guangzhou, China).

**TABLE 1:** Collection sites of *Rhododendron cyanocarpum* on the Cangshan Mountains, Yunnan, China, including population id, location name, coordinates, and sample size (number of individuals used in the analysis).

Pop ID	Location name	Sample size	Latitude	Longitude	Altitude (m)
HDB	Huadianba	15	99°59′09.65′′	25°52′04.69′′	3,341
XHD	Xiaohuadianba	15	100°03′15.76′′	25°52′35.95′′	3,442
DST	Dianshita	14	100°05′44.80′′	25°40′35.85′′	3,796
DHY	Dahuayuan	15	100°00′33.93′′	25°47′35.61′′	3,447

### ddRAD Library Preparation and Sequencing

Library preparation was conducted following Peterson et al. (2012). The genomic DNA (100 ng 10 μl) was double digested using 10 μl of the restriction enzymes EcoR I and Mse I for 5 h at 37°C, then 20 min at 65°C, and final incubation at 12°C. The resulting digested fragments were cleaned and subsequently quantified using agarose gel electrophoresis. Digested fragments were ligated to EcoR I and Mse I adapters containing sample specific barcodes with T4 DNA ligase (NEB) for 4 h at 16°C, then 20 min at 65°C, and final incubation at 12°C. Individually barcoded samples were cleaned and size-selected (350–500 bp) using agarose gel (Omega kit). Each library was then PCR-amplified to the desired concentration and paired-end sequenced (0.5 G each sample) on an Illumina X-ten (Illumina) with PE 150 mode.

### SNP Calling

Quality filtering and locus assembly were conducted using the Stacks software, version 2.4 (Catchen et al., 2013). RAD-tags were demultiplexed using process_radtags, the len_limit was set as 140 bp to trim low-quality reads, and retain_header -t was set to 135. We then used ustacks (parameter as follows: –min depth of coverage to create a stack (m): 2, –repeat removal algorithm: enabled, –max distance allowed between stacks (M): 2, –max distance allowed to align secondary reads: 4, –max number of stacks allowed per *de novo* locus: 3, –deleveraging algorithm: disabled, –gapped assembly: enabled, –minimum alignment length: 0.8, –model type: SNP, –alpha significance level for model: 0.05) to cluster and generate loci. All the loci were merged into the catalog using cstacks with the default parameters. The populations program (–min-populations: 4, –min-samples-per-pop: 0.8, –max-obs-het: 0.6, –write-single-snp) was used to call the SNPs across all ddRAD sites, the parameter “–write-single-snp” was used to restrict data analysis to only the first SNP per locus (Catchen et al., 2013; O’Leary et al., 2018).

### Analysis of Genetic Diversity and Structure

Tajima’s D was calculated in vcftools v.0.1.16 (Danecek et al., 2011) with 95% confidence limit (−1.795 to 2.052) to test all the loci for neutrality (Tajima, 1989b; Anderson et al., 2016). Window size for vcftools in calculating the Tajima’s D statistic was set as 3,000 bp. The loci were then divided into two sets: neutral loci and non-neutral loci evolving under putative selection. BayeScan v.2.1 was also used to infer loci under selection in each of the four sampled populations (Foll and Gaggiotti, 2008). PGDSpider v.2.1.1.5 was used for format conversion for the subsequent analysis (Lischer and Excoffier, 2011).

Population genetic statistics, including the number of private alleles, heterozygosity (*H*_*o*_), nucleotide diversity (π), and Wright’s F statistics *F*_*st*_ and *F*_*IS*_ statistics, were calculated using the “populations” program in Stacks (Catchen et al., 2013), and were tested for significance among populations by SPSS 16.0 software. Normality and variance homogeneity were first tested in all loci (26,336 observations), neutral loci (22,916 observations), and selected loci (3,420 observations) independently; because they violated the assumption of One-way ANOVA, Nonparamatric Tests were performed among populations. Geographic distance was calculated between each pair of sample locations using scripts from the Movable Type Scripts webpage^1^.

To examine the population structure of *R. cyanocarpum*, a Bayesian-based analysis was performed using the software Structure v.2.3 (Pritchard et al., 2000), with a burn-in of 1,000 steps and 5,000 replicates for each value of K. The optimal K for each analysis was chosen using Harvester v.0.694^2^. Genetic relationships among the studied individuals were also assessed with a principal component analysis (PCA) performed using Plink v.1.9 (Purcell et al., 2007) and R v.3.6.1 (R Development Core Team, 2013) to identify the population structure and to show the first two major axes explaining genetic variation. For this analysis, a variant call format (VCF) file was also generated using the “populations” program in Stacks. An analysis of molecular variance (AMOVA) with 1,000 permutations for each population using all loci, neutral loci, and selected loci were performed separately in the program Arlequin v.3.5 (Excoffier and Lischer, 2010) to calculate pairwise *F*_*st*_-values, with statistically significant differentiation being determined using a *p*-value of <0.05.

### Demographic History

To investigate the recent demographic history of *R. cyanocarpum*, we used the python script easySFS^3^ to generate the folded site frequency spectrum (SFS) formatted file with VCF files, and the demographic history was then inferred using the program Stairway plot v.0.2 (Liu and Fu, 2015) based on neutral loci with the recommended 67% of sites for training. Stairway plot is based on the site frequency spectrum (SFS) that does not require whole-genome sequence data or a reference genome, and is more accurate for inferring recent population size changes compared to the PSMC or MSMC methods (Liu and Fu, 2015). However, this method can be affected by the estimation of mutation rate (Lapierre et al., 2017). Patton et al. (2019) used four methods to infer the history of population size from genomic datasets and compared their performance. Their results suggested that the Stairway Plot has the most accurate estimation of the shape of recent trends, but that it tends to underestimate contemporary population sizes.

Cross (1975) observed that *R. ponticum* began to flower at about 12 years old; Tamaki et al. (2017) gave the generation time for *R. japonoheptamerum* as 30 years, as they described that *R. japonoheptamerum* is a shrub with a very low growth rate. To infer the recent demographic history, we therefore set the generation time for *R. cyanocarpum* to 10, 20, and 30 years per generation. The mutation rate of *R. weyrichii* had been determined to be 1.581 × 10^–9^ per site per year by Yoichi et al. (2016), and we therefore set a mutation rate of 1.581 × 10^–8^ per site per generation (generation time = 10), 3.162 × 10^–8^ per site per generation (generation time = 20) and 4.743 × 10^–8^ per site per generation (generation time = 30) for *R. cyanocarpum*.

## Results

### Sequence Data Quality and Processing

After all quality filters, 3,337,583 reads with no rad tag and 12,793 reads with low quality were discarded. We retained a total of 344,001,104 reads from the initial 347,351,480 raw reads, with an average of 5,830,527 reads per sample. After trimming and clustering, we obtained 9,148,349 loci. Mean locus coverage across all samples was 23.91×, ranging from 17.77× to 58.41×, with an average length of 136 bp per loci and with 40–48% GC content. After the “cstacks” module process, we obtained 2,464,683 catalogs (Supplementary Table S1). Finally, 6,584 SNPs were retained.

### Genetic Diversity and Population Structure

After filtering using Tajima’s D in Vcftools, a total of 855 SNPs evolving non-randomly (”selected”), and 5,729 SNPs evolving randomly (”neutral”) were retained. The Tajima’s D value for the whole dataset at the species level was –0.2748. Significantly negative Tajima’s D values were found, indicating that the haplotype frequencies deviated from the neutrality model (Tajima, 1989b). Selected loci and neutral loci were also tested based on Bayescan v.2.1. Only two loci were detected to be under selection (*q* < 0.05) with this method. Hence, our final datasets for further analysis were all loci (6,582 neutral SNPs identified by Bayescan), neutral loci (5,729), and selected loci (855) identified by Tajima’s D.

The selected loci dataset has the lowest number of private alleles in each population (157–221), neutral loci dataset is in the middle (911–1136), and all loci dataset has the highest number of private alleles (1081–1351). The genetic parameters *H*_*e*_ and π showed significant difference among the four populations for all loci (*H*_*o*_: *p* = 0.004; *H*_*e*_: *p* = 0.000; π: *p* = 0.000; *F*_*IS*_: *p* = 0.001), neutral loci (*H*_*o*_: *p* = 0.049; *H*_*e*_: *p* = 0.000; π: *p* = 0.000; *F*_*IS*_: *p* = 0.001), and selected loci (*H*_*o*_: *p* = 0.001; *H*_*e*_: *p* = 0.000; π: *p* = 0.000; *F*_*IS*_: *p* = 0.074). The inbreeding coefficients were positive in the four populations, reflecting a deficit of heterozygotes (inbreeding) (Boscari et al., 2019). The summary statistics of genetic diversity using all loci, neutral loci, and selected loci are given in Table 2.

**TABLE 2:** Population genetic statistics of *R. cyanocarpum* on the Cangshan Mountains, Yunnan, China: observed heterozygosity (*H*_*o*_), expected heterozygosity (*H*_*e*_), genetic diversity (π), inbreeding coefficients (*F*_*IS*_).

	Pop ID	Private alleles	*H*_*o*_	*H*_*e*_	π	*F*_*IS*_
**All loci**						
	HDB	1192	0.0430 ± 0.0011	0.0615 ± 0.0014	0.0639 ± 0.0015	0.0772 ± 0.0120
	XHD	1081	0.0408 ± 0.0011	0.0609 ± 0.0015	0.0634 ± 0.0015	0.0800 ± 0.0114
	DST	1350	0.0414 ± 0.0010	0.0637 ± 0.0015	0.0664 ± 0.0015	0.0921 ± 0.0093
	DHY	1351	0.0426 ± 0.0010	0.0657 ± 0.0015	0.0683 ± 0.0015	0.0952 ± 0.0129
	Mean	1244	0.0420 ± 0.0011	0.0630 ± 0.0015	0.0655 ± 0.0015	0.0861 ± 0.0114
**Neutral loci**						
	HDB	1035	0.0456 ± 0.0012	0.0663 ± 0.0016	0.0689 ± 0.0017	0.0843 ± 0.0129
	XHD	911	0.0432 ± 0.0012	0.0656 ± 0.0016	0.0682 ± 0.0017	0.0867 ± 0.0121
	DST	1129	0.0435 ± 0.0012	0.0680 ± 0.0016	0.0708 ± 0.0017	0.0983 ± 0.0100
	DHY	1136	0.0443 ± 0.0011	0.0703 ± 0.0016	0.0730 ± 0.0017	0.1053 ± 0.0138
	Mean	1053	0.0442 ± 0.0012	0.0675 ± 0.0016	0.0702 ± 0.0017	0.0936 ± 0.0122
**Selected loci**						
	HDB	157	0.0251 ± 0.0023	0.0295 ± 0.0024	0.0306 ± 0.0025	0.0295 ± 0.0335
	XHD	170	0.0245 ± 0.0022	0.0301 ± 0.0024	0.0312 ± 0.0025	0.0350 ± 0.0329
	DST	221	0.0274 ± 0.0021	0.0351 ± 0.0024	0.0365 ± 0.0025	0.0508 ± 0.0262
	DHY	215	0.0311 ± 0.0023	0.0348 ± 0.0024	0.0362 ± 0.0025	0.0278 ± 0.0357
	Mean	191	0.0270 ± 0.0022	0.0323 ± 0.0024	0.0336 ± 0.0025	0.0358 ± 0.0320

The pairwise *F*_*st*_ values between populations were less than 0.05 (Table 3), indicating that all populations of *R. cyanocarpum* were genetically similar and there was little differentiation among them (Ewens, 1969), which was also supported by the analysis of molecular variance (AMOVA, Table 4). The global AMOVA revealed that most genetic variation was found within populations, with very little among populations.

**TABLE 3:** Genetic distances (*F*_*st*_ values, above diagonal) and geographic distances (km, below diagonal) between *R. cyanocarpum* populations on the Cangshan Mountains, Yunnan, China.

	HDB	XHD All/Neutral/Selected	DST All/Neutral/Selected	DHY All/Neutral/Selected
HDB	–	0.0306/0.0314/0.0240	0.0427/0.0444/0.0292	0.0338/0.0351/0.0232
XHD	6.90	–	0.0432/0.0452/0.0279	0.0332/0.0346/0.0226
DST	23.93	22.61	–	0.0361/0.0376/0.0256
DHY	8.63	10.30	15.58	–

**TABLE 4:** AMOVA results: evaluation of genetic differentiation within and among sampling sites of *R. cyanocarpum* on the Cangshan Mountains, Yunnan, China.

Source of variation	Sum of squares All/Neutral/Selected	Variance components All/Neutral/Selected	Percentage variation (%) All/Neutral/Selected	F-Statistics All/Neutral/Selected
Among populations	2883.184/2588.350/294.834	10.584/9.833/0.701	1.60/1.70/0.90	0.0160/0.0170/0.0170
Within populations	73976.274/65128.726/8847.548	648.915/571.305/77.610	98.40/98.30/99.11	
Total	76859.458/67717.076/9142.381	659.499/581.188/78.310		
				

**FIGURE 2:**
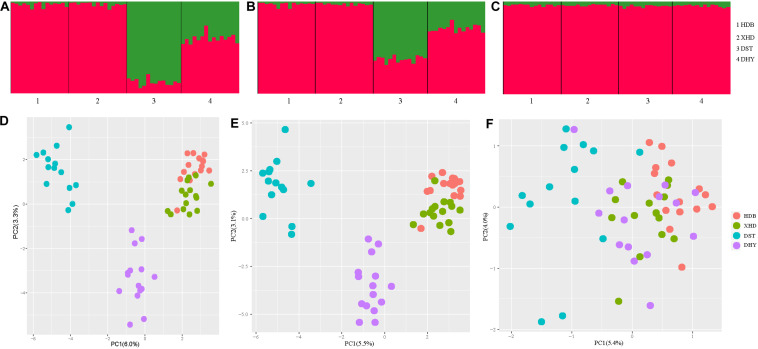
Population structure of *R. cyanocarpum* on the Cangshan Mountains, Yunnan, China. **(A–C)** Structure results (*K* = 2), with different colors showing different genetic backgrounds [**(A)** using all loci, **(B)** using neutral loci, **(C)** using selected loci]; **(D–F)** results of principal components analysis [**(D)** using all loci, **(E)** using neutral loci, **(F)** using selected loci].

Δ*K* = 2 was best supported for all three types of loci (Supplementary Figure S1). Similar patterns of population structure were inferred from both all loci and neutral loci, while DST had fewer admixture characteristics deduced from all loci (an average of 88% green vs. 12% red genetic backgrounds) than neutral loci (an average of 60% green vs. 40% red genetic backgrounds). Two clear genetic clusters are visible from the Structure results using all loci (Figure 2A) and neutral loci (Figure 2B), with populations HDB and XHD belonging to cluster 1, and populations DST and DHY belonging to cluster 2. However, the pattern revealed from selected loci differed from the other two types of loci, as only one genetically homogeneous group was detected (Figure 2C). Similar patterns were suggested using principal coordinate analysis. Three clusters were detected using all loci (Figure 2D) and neutral loci (Figure 2E), one for HDB and XHD, and one for DST and one for DHY. Additionally, the four populations were clustered to one group using PCA based on selected loci (Figure 2F).

### Effective Population Size and Demographic History

**FIGURE 3:**
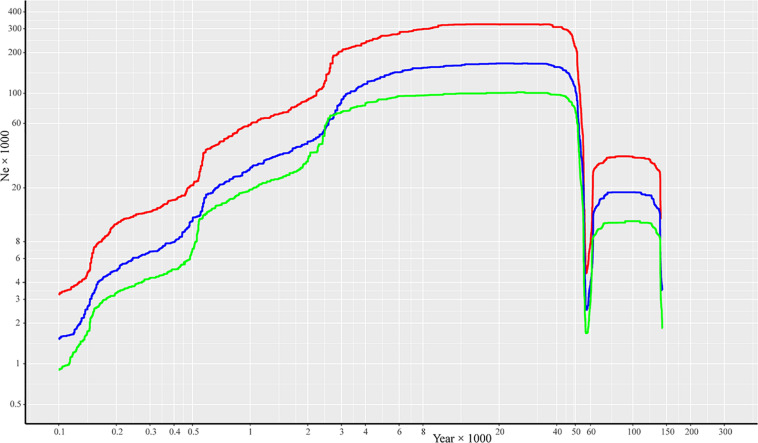
Reconstructing the demographic history of *R. cyanocarpum* on the Cangshan Mountains, Yunnan, China. Stairway plot showing historical changes in effective population size (*N*_*e*_ × 1000, y-axis) for *R. cyanocarpum* population with generation times of 10 years (red line), 20 years (blue line), and 30 years (green line).

Effective population size and demographic history of *R. cyanocarpum* were inferred based on SNP frequency spectra and displayed on a stairway plot. A population bottleneck occurred at ca. 0.06 Ma (million years ago), after which followed a population expansion and then a gradual reduction in population size to ca. 100 years ago (Figure 3). Despite the differences observed in the effective population size, we found similar demographic patterns when using generation times of 30, 20, and 10 years.

## Discussion

### Genetic Diversity

It is generally believed that characterizing genetic diversity and population structure are essential for the effective conservation of threatened species. The genetic diversity of *R. cyanocarpum* (0.0634–0.0683 with all loci, 0.0682–0.0730 with neutral loci) was higher compared with previous studies on other plant species using ddRAD sequencing. For example, a much lower level was found in endangered *Viola uliginosa* (0.013–0.023) (Lee et al., 2020), *Clermontia fauriei* (0.0014), and *Cyanea pilosa* (0.0012) populations (Jennings et al., 2016); we are not aware of other studies on rhododendrons using ddRAD-seq. We can therefore conclude that genetic diversity in *Rhododendron cyanocarpum* is higher despite its small population size. The higher genetic diversity seen in *R. cyanocarpum* could be due to its population demography. As a woody species growing in an alpine habitat, *R. cyanocarpum* is likely to have a long lifespan; for example, the generation time of *R. japonoheptamerum* is about 30 years (Tamaki et al., 2017). It has been recorded that ca. 60 years ago, many *R. cyanocarpum* trees were cut down to aid the building of the Huadianba Medical Factory (Ma et al., 2010a), close to the HDB and XHD populations, which probably resulted in a recent reduction in population size of *R. cyanocarpum*. In such a scenario, however, we would expect that *R. cyanocarpum* would still maintain a similar amount of genetic diversity as before, due to the very limited role played by genetic drift over such a short time period (Luan et al., 2006).

### Genetic Structure and Genetic Differentiation

Gene flow is a main factor affecting genetic differentiation between populations. The low genetic differentiation among populations of *R. cyanocarpum* could be explained by gene flow resulting from long-distance gene dispersal either as pollen or as seeds (Zhao et al., 2012). All of the study populations of *R. cyanocarpum* were close geographically, with a distance range of 6.9–23.9 km, which is beneficial for gene flow facilitated by pollinators and seed dispersal. Previous studies suggested that birds were another important pollinator in *R. cyanocarpum* (Ma et al., 2015a), which can favor pollen flow between populations because of birds’ long-distance flight. *Rhododendron* seeds, characterized as small, light and winged, are frequently dispersed by wind (Ng and Corlett, 2000).

For the results using Structure and PCA, similar patterns were detected based on selected loci and neutral loci in *R. cyanocarpum.* When using all loci or neutral loci, the results of Structure and PCA revealed two or three distinct groups in *R. cyanocarpum* populations, respectively. In contrast, the patterns of population structure resulting from the selected loci dataset showed no distinct subgroups, which could be explained by natural selection. At small spatial scales, local environmental conditions often shape the patterns of genetic structure, especially in heterogeneous and fragmented habitats (Young et al., 1996; Zhu et al., 2016). While there are distances of 6–23 km among the populations, a valley within the mountain range, as well as other topographic variation, may represent stabilizing selection for *R. cyanocarpum* adaptation to shape the patterns of genetic structure.

### Effective Population Size and Demographic History

According to our stairway plot to illustrate the demographic history of *R. cyanocarpum*, a population bottleneck occurred at about 0.058 Ma, which was consistent with the late Pleistocene extinction (ca. 0.05 Ma) (Stuart, 1999; Van Der Kaars et al., 2017), and was also consistent with the process termed the early Dali Glaciation (0.058 Ma), when the estimated snow line was lower than it currently is, glacial and periglacial processes were taking place in areas above 3,500 m (Yang et al., 2006; Wan et al., 2011), and the temperatures were lower than at present. Extremely low temperatures may seriously damage and inhibit growth of *R. cyanocarpum*. During our field investigations, we saw that *R. cyanocarpum* grew up to altitudes of about 3,860 m.

The stairway plot also suggested that *R. cyanocarpum* underwent a population expansion in the period before and after the Last Glacial Maximum (LGM, 0.04–0.006 Ma) (Garot et al., 2019). That this population expansion event took place was also supported by the negative value of Tajima’s D (–0.2748 with 95% confidence level), because strong negative values of Tajima’s D can indicate population expansions (Tajima, 1989a). Recently, over the last 7,000 years, the *N*_*e*_ of *R. cyanocarpum* has been declining, which is consistent with environmental exploitation, deforestation, clearance of forested areas, and other intensive human activities. The first evidence for anthropogenic disturbance on the Cangshan Mountains is from ca. 6,370 years ago based on pollen records, when forests were being cleared for shifting agriculture (Shen et al., 2006). Together with climatic warming, this deforestation could have led to the decline in the *R. cyanocarpum* populations. Further evidence of disturbance caused by anthropogenic activities on the Cangshan Mountains stems from Neolithic ruins (about 3,100 years ago), the Bronze Age (from 2,000 years ago) (Wan et al., 2011), the inward migration and establishment of the Han Chinese (about 2,300–1,700 years ago), and the ancient city of Nanzhao and the Dali Kingdom (from 1,000 years ago) (Dearing et al., 2008). Taken together, frequent anthropogenic activities, such as agriculture, pastoralism, irrigation, vegetation clearance and deforestation, and burning and grazing, as well as the onset of warmer summers (Dearing et al., 2008), all posed great threats to *R. cyanocarpum* and resulted in reduction of effective population sizes.

### Implications for Conservation and Management of *R. cyanocarpum*

*R. cyanocarpum* underwent a bottleneck, a subsequent expansion, and a gradual reduction. With intensive anthropogenic activity and global warming, the species has become fragmented and now has a highly restricted range near the summit of the Cangshan Mountains. Based on our main findings, we recommend the following conservation measures for *R. cyanocarpum*. (i) Although all populations are located in a national nature reserve, habitat disturbance from anthropogenic activity still occurs frequently, including tourism, path construction, and pasture and land reclamation. Absolute prohibition of anthropogenic activities is not possible and is also not necessary, although in the national nature reserve deforestation and large-scale land reclamation should be banned absolutely, especially near the HDB and XHD populations, where there is a big pharmaceutical farm. (ii) Because most individuals of *R. cyanocarpum* are situated on ridges of the Cangshan Mountains, and no distinct subgroups were detected when using selected loci, we suppose that the most serious threat to *R. cyanocarpum* will be not anthropogenic activities but instead be global warming. Given the topographic and landscape constraints, the species has limited migration potential, and *ex situ* conservation is therefore urgent and should be conducted immediately, including seed collection and conservation, and searching for new suitable habitats. (iii) The DHY population had the highest level of genetic diversity of the four populations, and we observed more connectivity and admixture between DHY and other populations than between any of the others, suggesting its importance in maintaining genetic polymorphism. This population is geographically located in the middle of other populations, which is beneficial for facilitating dispersal and connecting the other populations as a corridor. Therefore, we suggest that more conservation effort be given to the population DHY.

## Data Availability Statement

The raw sequencing data has been successfully uploaded to SRA in NCBI, https://www.ncbi.nlm.nih.gov/Traces/study/?acc=PRJNA640883.

## Author Contributions

YM conceived and designed the experiments and revised the manuscript. LZ carried out investigation and collected materials. DL analyzed the data and wrote the manuscript. YM and JW acquired the funding. All authors contributed to the article and approved the submitted version.

## Conflict of Interest

The authors declare that the research was conducted in the absence of any commercial or financial relationships that could be construed as a potential conflict of interest.

## Footnotes

1 http://www.movable-type.co.uk/scripts/latlong.html

2 http://taylor0.biology.ucla.edu/structureHarvester

3 https://github.com/isaacovercast/easySFS
