# Supplementary figures and images for: Conservation Genomics of a Threatened Rhododendron: Contrasting Patterns of Population Structure Revealed From Neutral and Selected SNPs
Source: Front Genet. 2020 Sep 4;11:757. doi: 10.3389/fgene.2020.00757 (PMC7500208; doi:10.3389/fgene.2020.00757)

$$\text{DeltaK} = \text{mean}(|L''(K)|) / \text{sd}(L(K))$$

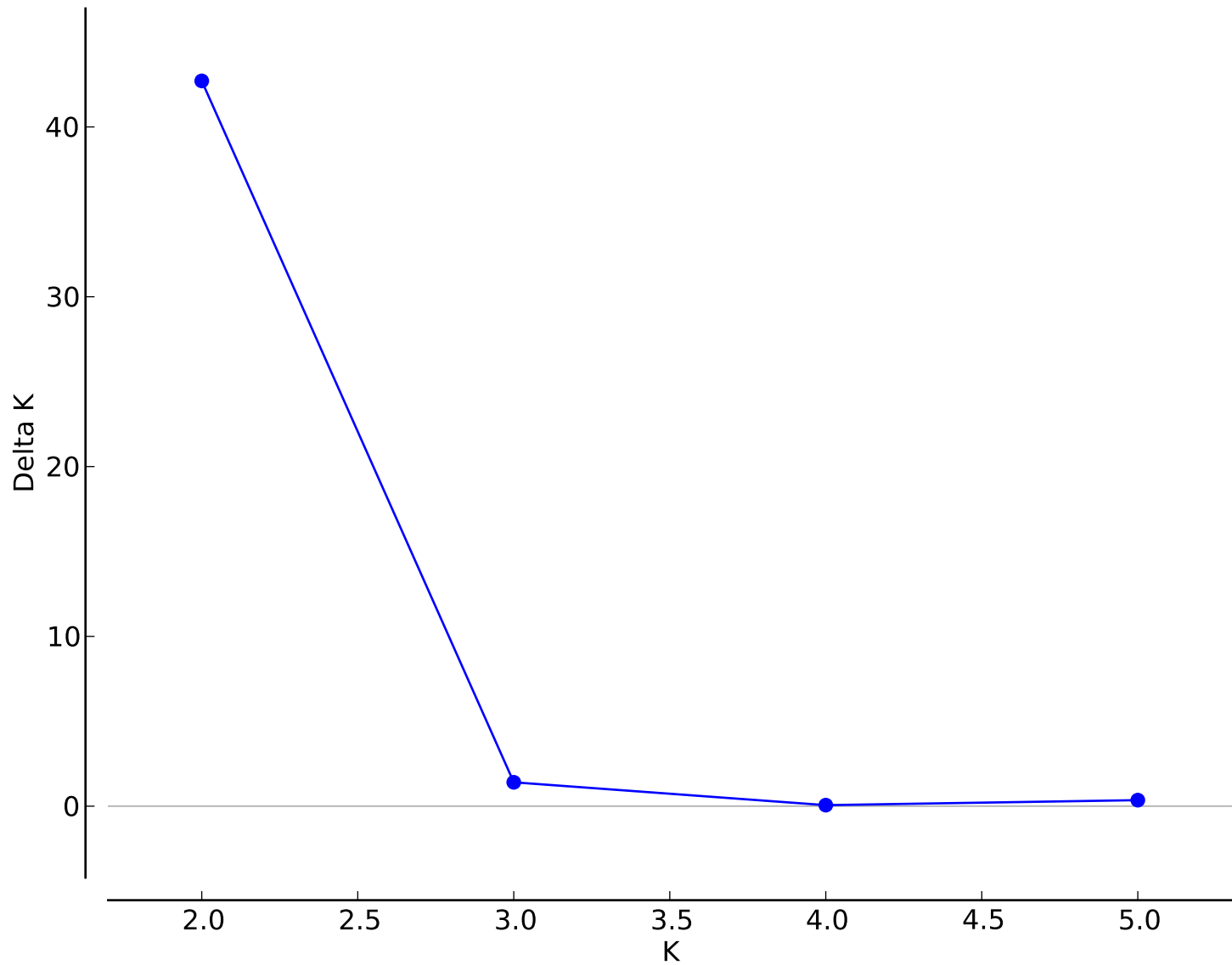

Supplement: Supplementary file 2 [file Data_Sheet_1.ZIP › Supplementary S2/deltaK_all loci.pdf]

$$\text{DeltaK} = \text{mean}(|L''(K)|) / \text{sd}(L(K))$$

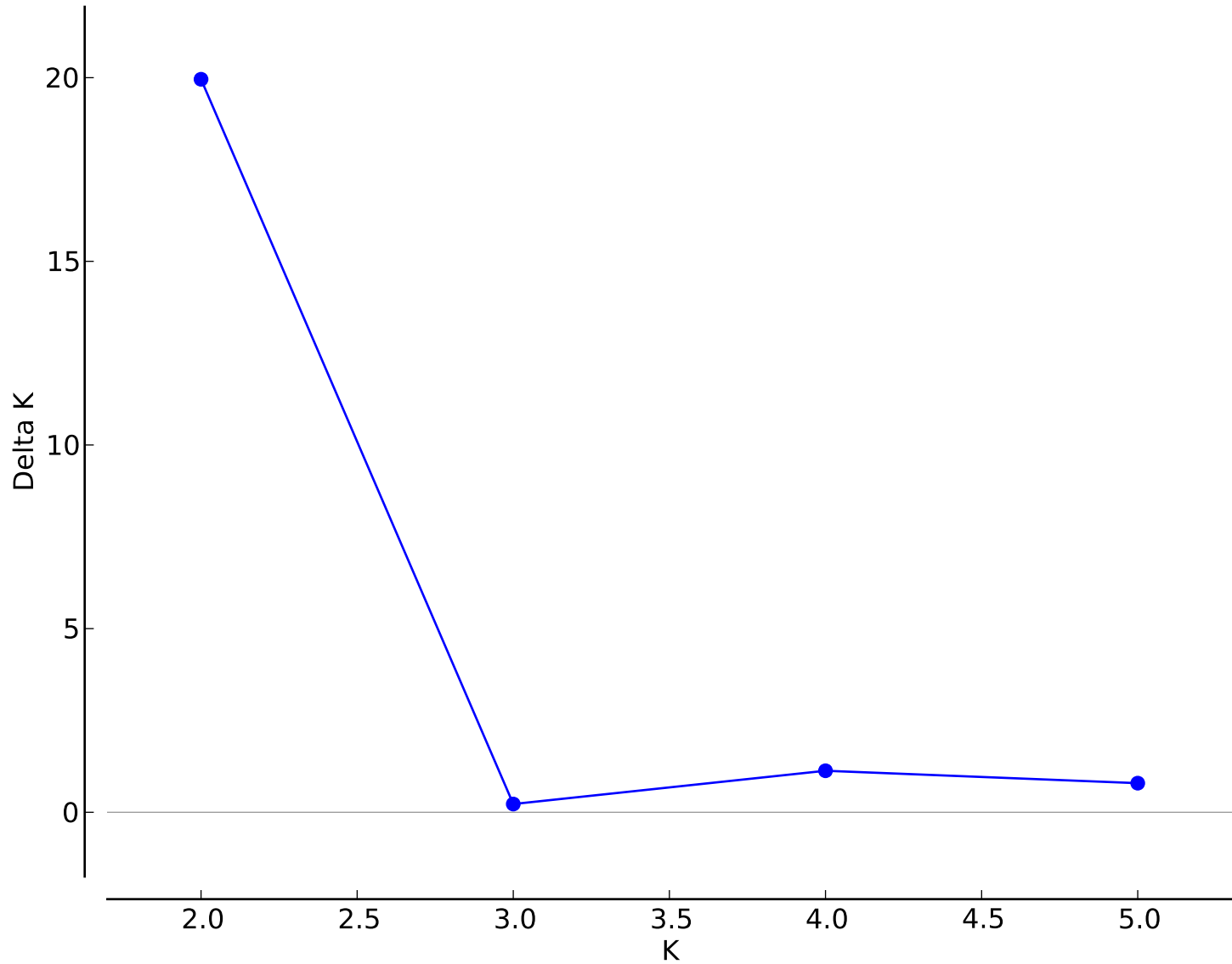

Supplement: Supplementary file 2 [file Data_Sheet_1.ZIP › Supplementary S2/deltaK_neutral loci.pdf]

$$\text{DeltaK} = \text{mean}(|L''(K)|) / \text{sd}(L(K))$$

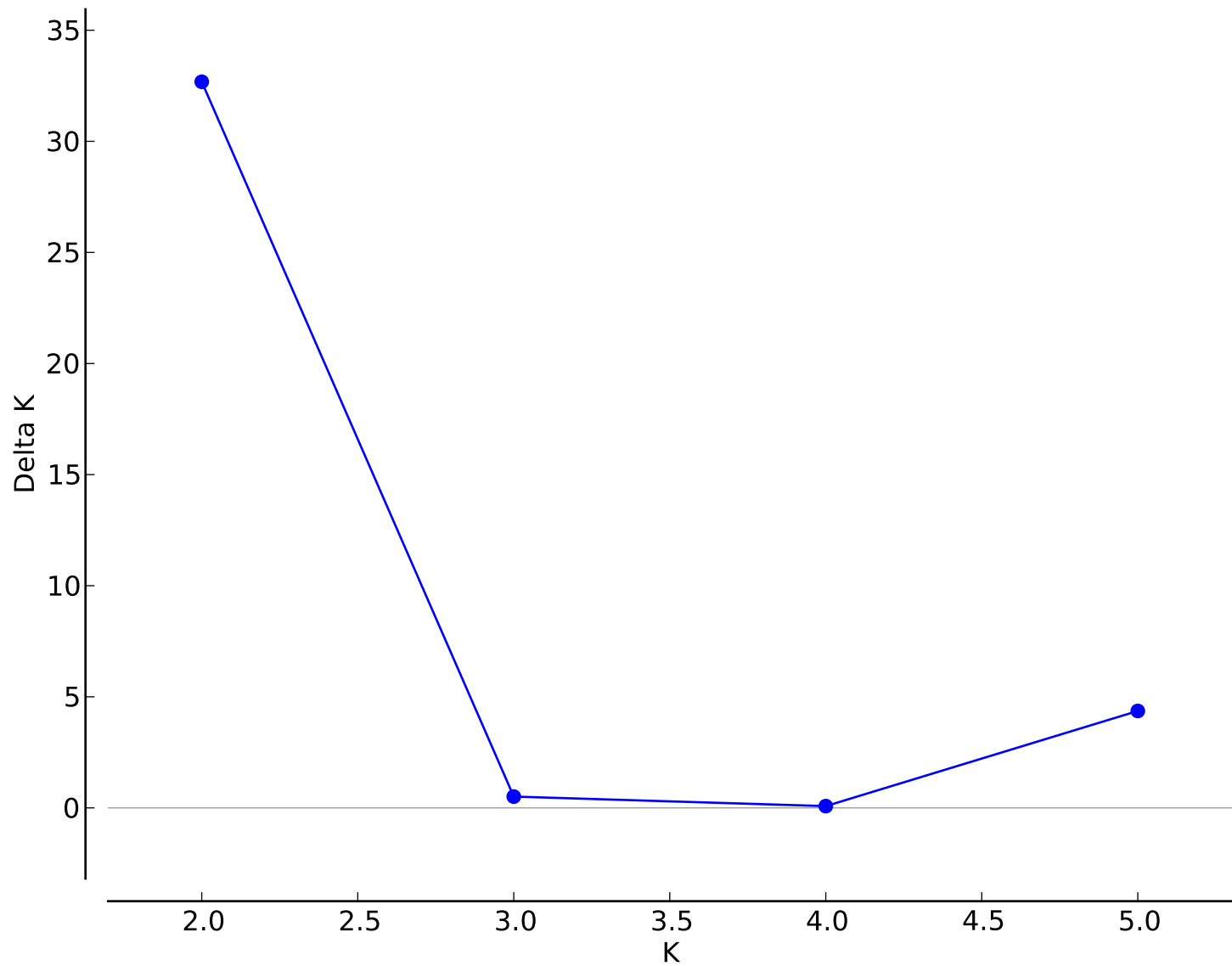

Supplement: Supplementary file 2 [file Data_Sheet_1.ZIP › Supplementary S2/deltaK_selected loci.pdf]
